# Supplementary material for: A psychometric evaluation of the Chinese Impact of Vision Impairment (C-IVI) questionnaire in an adult cohort with high myopia using Rasch analysis
Source: PLoS One. 2025 Oct 9;20(10):e0327708. doi: 10.1371/journal.pone.0327708 (PMC12510582; doi:10.1371/journal.pone.0327708)
Supplement: S1 Table — (DOCX) [file pone.0327708.s001.docx]

| **S1 Table:** Structure, Item Content, and Domain of the C-IVI questionnaire | |
| --- | --- |
| **Item** | **Summary domains** |
| 1.Ability to see and enjoy television? | Reading and accessing information |
| 3.Finding things during shopping? |  |
| 5.Recognizing or meeting people? |  |
| 6.Generally looking after your appearance? |  |
| 7.Opening packaging? |  |
| 8.Reading labels or instructions on medicines? |  |
| 9.Operating household appliances and the telephone? |  |
| 14.Reading ordinary-sized print? |  |
| 15.Getting information that you need? |  |
| 2.Taking part in sporting activities? | Mobility and independence |
| 4.Vising friends or family? |  |
| 10.Getting about outdoors |  |
| 11.Made you go carefully to avoid falling or tripping? |  |
| 12.Interfered with travelling or using transport? |  |
| 13.Going down steps, stairs, or curbs? |  |
| 16.Your general safety at home? |  |
| 17.Spilling or breaking things? |  |
| 18.Your general safety when out of your home? |  |
| 19.Stopped you from doing the things you want to do? |  |
| 20.Needed help from other people? |  |
| 21.Felt embarrassed? | Emotional well -being |
| 22.Felt frustrated or annoyed? |  |
| 23.Felt lonely or isolated? |  |
| 24.Felt sad or low? |  |
| 25.Worried about your eyesight getting worse? |  |
| 26.Concerned or worried about coping with everyday life? |  |
| 27.Felt like a nuisance or a burden? |  |
| 28.Interfered with your life in general? |  |
