# Supplementary material for: A psychometric evaluation of the Chinese Impact of Vision Impairment (C-IVI) questionnaire in an adult cohort with high myopia using Rasch analysis
Source: PLoS One. 2025 Oct 9;20(10):e0327708. doi: 10.1371/journal.pone.0327708 (PMC12510582; doi:10.1371/journal.pone.0327708)
Supplement: S2 Table — (DOCX) [file pone.0327708.s002.docx]

| **S2 Table**: Item Frequency Table of the C-IVI 28 | | |
| --- | --- | --- |
| **Item categories** | **Data Count (N%)** | **Average Ability** |
| Item-1 |  |  |
| Missing | 8 (2%) | 3.87 |
| 0 | 3 (1%) | 1.19 |
| 1 | 27 (6%) | 1.16* |
| 2 | 158 (37%) | 2.78 |
| 3 | 235 (56%) | 4.83 |
| Item-2 |  |  |
| Missing | 14 (3%) | 3.02 |
| 0 | 3 (1%) | 0.31 |
| 1 | 14 (3%) | 1.07 |
| 2 | 108 (26%) | 2.18 |
| 3 | 292 (70%) | 4.61 |
| Item-3 |  |  |
| Missing | 5 (1%) | 2.1 |
| 0 | 2 (0%) | 0.67 |
| 1 | 13 (3%) | .56* |
| 2 | 85 (20%) | 1.99 |
| 3 | 326 (77%) | 4.45 |
| Item-4 |  |  |
| Missing | 4 (1%) | 1.96 |
| 0 | 2 (0%) | -0.01 |
| 1 | 2 (0%) | -.26* |
| 2 | 55 (13%) | 1.35 |
| 3 | 368 (86%) | 4.24 |
| Item-5 |  |  |
| Missing | 2 (0%) | 2.4 |
| 0 | 2 (0%) | 0.95 |
| 1 | 23 (5%) | .92* |
| 2 | 94 (22%) | 2.26 |
| 3 | 310 (72%) | 4.52 |
| Item-6 |  |  |
| Missing | 4(1%) | 2.84 |
| 0 | 3(1%) | 0.22 |
| 1 | 27(6%) | 1.35 |
| 2 | 113(26%) | 2.46 |
| 3 | 284(67%) | 4.63 |
| Item-7 |  |  |
| Missing | 2(0%) | 2.4 |
| 1 | 2(0%) | -0.53 |
| 2 | 64(15%) | 1.37 |
| 3 | 363(85%) | 4.27 |
| Item-8 |  |  |
| Missing | 3(1%) | 3.83 |
| 1 | 17(4%) | 0.84 |
| 2 | 122(29%) | 2.42 |
| 3 | 289(68%) | 4.57 |
| Item-9 |  |  |
| Missing | 2(0%) | 2.4 |
| 1 | 4(1%) | 0.4 |
| 2 | 62(14%) | 1.35 |
| 3 | 363(85%) | 4.27 |
| Item-10 |  |  |
| Missing | 4(1%) | 2.47 |
| 0 | 3(1%) | 0.83 |
| 1 | 14(3%) | .50* |
| 2 | 132(31%) | 2.34 |
| 3 | 278(65%) | 4.72 |
| Item-11 |  |  |
| Missing | 1(0%) | 2.23 |
| 0 | 1(0%) | -0.17 |
| 1 | 12(3%) | 0.27 |
| 2 | 90(21%) | 2 |
| 3 | 327(76%) | 4.45 |
| Item-12 |  |  |
| Missing | 4(1%) | 2.31 |
| 0 | 2(0%) | -0.16 |
| 1 | 19(4%) | 0.99 |
| 2 | 138(32%) | 2.49 |
| 3 | 268(63%) | 4.74 |
| Item-13 |  |  |
| Missing | 3(1%) | 1.74 |
| 0 | 2(0%) | -0.39 |
| 1 | 11(3%) | 0.58 |
| 2 | 90(21%) | 1.97 |
| 3 | 325(76%) | 4.47 |
| Item-14 |  |  |
| Missing | 6(1%) | 1.95 |
| 0 | 2(0%) | 0.63 |
| 1 | 97(23%) | 1.82 |
| 3 | 326(77%) | 4.45 |
| Item-15 |  |  |
| Missing | 10(2%) | 1.72 |
| 0 | 4(1%) | 0.57 |
| 1 | 57(14%) | 1.15 |
| 3 | 360(86%) | 4.32 |
| Item-16 |  |  |
| 1 | 6(1%) | 0.7 |
| 2 | 19(4%) | 1.21 |
| 3 | 406(94%) | 3.97 |
| Item-17 |  |  |
| 1 | 6(1%) | 0.19 |
| 2 | 28(6%) | 1.38 |
| 3 | 397(92%) | 4.03 |
| Item-18 |  |  |
| 1 | 7(2%) | 0.73 |
| 2 | 29(7%) | 1.33 |
| 3 | 395(92%) | 4.04 |
| Item-19 |  |  |
| 0 | 2(0%) | -0.57 |
| 1 | 43(10%) | 1.43 |
| 2 | 74(17%) | 2.31 |
| 3 | 312(72%) | 4.52 |
| Item-20 |  |  |
| 1 | 14(3%) | 0.68 |
| 2 | 51(12%) | 1.71 |
| 3 | 366(85%) | 4.22 |
| Item-21 |  |  |
| 0 | 5(1%) | 0.21 |
| 1 | 48(11%) | 1.4 |
| 2 | 105(24%) | 2.72 |
| 3 | 273(63%) | 4.71 |
| Item-22 |  |  |
| 0 | 8(2%) | 0.81 |
| 1 | 60(14%) | 1.7 |
| 2 | 117(27%) | 2.78 |
| 3 | 246(57%) | 4.91 |
| Item-23 |  |  |
| 0 | 2(0%) | -0.19 |
| 1 | 17(4%) | 0.41 |
| 2 | 38(9%) | 1.76 |
| 3 | 374(87%) | 4.19 |
| Item-24 |  |  |
| 0 | 5(1%) | 0.35 |
| 1 | 39(9%) | 1.41 |
| 2 | 91(21%) | 2.45 |
| 3 | 296(69%) | 4.6 |
| Item-25 |  |  |
| 0 | 13(3%) | 1.39 |
| 1 | 115(27%) | 2.38 |
| 2 | 125(29%) | 3.22 |
| 3 | 178(41%) | 5.32 |
| Item-26 |  |  |
| 0 | 6(1%) | 0.92 |
| 1 | 45(10%) | 1.44 |
| 2 | 73(17%) | 2.17 |
| 3 | 307(71%) | 4.6 |
| Item-27 |  |  |
| 0 | 6(1%) | 0.3 |
| 1 | 44(10%) | 1.96 |
| 2 | 66(15%) | 2.37 |
| 3 | 315(73%) | 4.43 |
| Item-28 |  |  |
| 0 | 4(1%) | -0.03 |
| 1 | 48(11%) | 1.45 |
| 2 | 112(26%) | 2.59 |
| 3 | 267(62%) | 4.8 |
| *Average ability does not ascend with category score | | |
