# Supplementary material for: A psychometric evaluation of the Chinese Impact of Vision Impairment (C-IVI) questionnaire in an adult cohort with high myopia using Rasch analysis
Source: PLoS One. 2025 Oct 9;20(10):e0327708. doi: 10.1371/journal.pone.0327708 (PMC12510582; doi:10.1371/journal.pone.0327708)
Supplement: S3 File — (DOCX) [file pone.0327708.s003.docx]

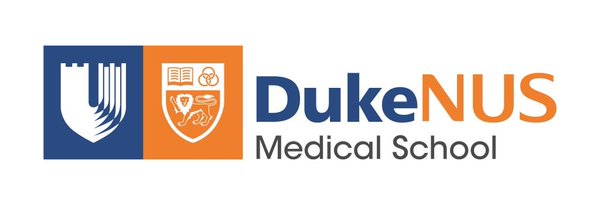

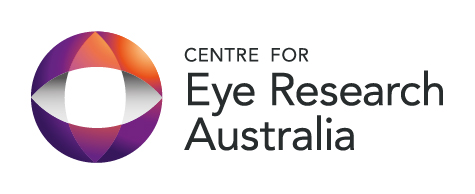

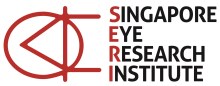


**视力受损的影响 （IVI）^TM^**

**Impact of Vision Impairment Profile (IVI)**

## 说明

请仔细阅读每一个问题，并圈出最适用于您答案。每行请只圈一处。

若您在进行某些活动时，有佩戴眼镜、隐形眼镜或使用放大镜，请回答以下问题根据您使用它们时的视力。

## INSTRUCTIONS

Please read each question carefully and circle the answer that BEST applies to you.

Put one circle on each row.

If you use GLASSES, CONTACT LENSES OR MAGNIFIERS for some activities please answer according to how you can see

when using them.

以下有两个例子：

在过去的一个月里，在进行以下活动时您有多经常因视力而感到关切或担心？

Here are two examples:

In the PAST MONTH, how often has YOUR EYESIGHT MADE YOU CONCERNED OR WORRIED about the following:

|  | 完全没有  Not at all | 有些时候  A little | 相当多的时候  A fair amount | 非常多的时候  A lot | 这件事与我无关  Don’t do this for other reasons |
| --- | --- | --- | --- | --- | --- |
| **过马路？** | **3** | **2** | **1** | **0** | **8** |
| **为自己准备一餐？** | **3** | **2** | 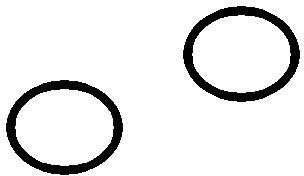**1** | **0** | **8** |

##
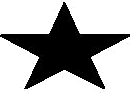
请从这里开始并记得：

每行请只圈一处。请不要少圈任何一行。

若有佩戴眼镜、隐形眼镜或使用放大镜， 请根据您使用它们时的视力回答以下问题。在过去的一个月内，您在进行这些活动时被视力干扰的程度有多大?

PLEASE START HERE AND REMEMBER:

Put one circle on each row. Please do not leave any rows blank.

Please answer about YOUR eyesight with GLASSES, CONTACT LENSES, or MAGNIFIERS, if you use them.

In the PAST MONTH, how much has YOUR EYESIGHT INTERFERED with the following activities:

|  | | 完全没有  Not at all | 有一点  A little | 相当多  A fair amount | 非常多  A lot | 这件事与我无关  Don’t do this for other reasons |
| --- | --- | --- | --- | --- | --- | --- |
| **1.** | **观赏电视节目？**  **Your ability to see and enjoy T.V.?** | **3** | **2** | **1** | **0** | **8** |
| **2.** | **参与休闲活动， 例如保龄球，散步或高尔夫？**  **Taking part in recreational activities such**  **as walking, jogging, bowling or golf?** | **3** | **2** | **1** | **0** | **8** |
| **3.** | **购物 (例如寻找及购买您要的东西）？**  **Shopping? (finding what you want and**  **paying for it)** | **3** | **2** | **1** | **0** | **8** |
| **4.** | **探访朋友或家人？**  **Visiting friends or family?** | **3** | **2** | **1** | **0** | **8** |
| **5.** | **识别他人或与人会面？**  **Recognising or meeting people?** | **3** | **2** | **1** | **0** | **8** |
| **6.** | **打理仪容（例如脸， 头发和穿着等）？**  **Generally looking after your appearance?**  **(face, hair, clothing, etc.)** | **3** | **2** | **1** | **0** | **8** |
| **7.** | **打开包装 （例如食物或药物的包装）？**  **Opening packaging? (for example, around**  **food, medicines)** | **3** | **2** | **1** | **0** | **8** |

若有佩戴眼镜、隐形眼镜或使用放大镜， 请根据您使用它们时的视力回答以下问题。在过去的一个月内，您在进行这些活动时被视力干扰的程度有多大?

Please answer about YOUR eyesight with GLASSES, CONTACT LENSES, or MAGNIFIERS, if you use them.

In the PAST MONTH, how much has YOUR EYESIGHT INTERFERED with the following activities:

|  | | 完全没有  Not at all | 有一点  A little | 相当多  A fair amount | 非常多  A lot | 这件事与我无关  Don’t do this for other reasons |
| --- | --- | --- | --- | --- | --- | --- |
| **8.** | **阅读药物标签或指示？**  **Reading labels or instructions on**  **medicines?** | **3** | **2** | **1** | **0** | **8** |
| **9.** | **使用家用电器和电话？**  **Operating household appliances and the**  **telephone?** | **3** | **2** | **1** | **0** | **8** |
| **10.** | **您在户外四处走动时（ 例如在行人道或过马路）被视力干扰的程度有多大？**  **How much has your eyesight interfered**  **with getting about outdoors? (on the**  **pavement or crossing the street)** | **3** | **2** | **1** | **0** | **8** |
| **11.** | **在过去的一个月内， 您多经常因为视力而需要特别小心以避免摔倒或绊倒？**  **In the past month, how often has your**  **eyesight made you go carefully to avoid**  **falling or tripping?** | **3** | **2** | **1** | **0** | **8** |
| **12.** | **一般情况下，您在外出时或使用公共交通时 （比如搭地铁或巴士等）被视力干扰的程度有多大？**  **In general, how much has your eyesight**  **interfered with travelling or using transport?**  **(bus & train)** | **3** | **2** | **1** | **0** | **8** |
| **13.** | **下梯级，楼梯或路肩？**  **Going down steps, stairs, or curbs?** | **3** | **2** | **1** | **0** | **8** |

若有佩戴眼镜、隐形眼镜或使用放大镜， 请根据您使用它们时的视力回答以下问题。在过去的一个月内，您在进行这些活动时被视力干扰的程度有多大?

Please answer about YOUR eyesight with GLASSES, CONTACT LENSES, or MAGNIFIERS, if you use them.

In the PAST MONTH, how much has YOUR EYESIGHT INTERFERED with the following activities:

|  | 完全没有  Not at all | 相当多  A fair amount | 非常多  A lot | 这件事与我无关  Don’t do this for other reasons |
| --- | --- | --- | --- | --- |
| 1. **阅读一般大小的字体（例如报纸）？**   **Reading ordinary size print? (for example newspapers)** | **2** | **1** | **0** | **8** |
| 1. **取得您所需要的资讯？**   **Getting information that you need?** | **2** | **1** | **0** | **8** |

若有佩戴眼镜、隐形眼镜或使用放大镜， 请根据您使用它们时的视力回答以下问题。在过去的一个月内，您多经常因为视力而担忧。。。?

Please answer about YOUR eyesight with GLASSES, CONTACT LENSES or MAGNIFIERS, if you use them.

In the PAST MONTH, how often has YOUR EYESIGHT MADE YOU CONCERNED OR WORRIED about the following:

|  | | 完全没有  Not at all | 有些时候  A little of time | 相当多的时候  A fair amount of time | 非常多的时候  A lot of time |
| --- | --- | --- | --- | --- | --- |
| **16.** | **您在家时的个人安危？**  **Your general safety at home?** | **3** | **2** | **0** | **0** |
| **17.** | **弄洒或打破东西？**  **Spilling or breaking things?** | **3** | **2** | **1** | **0** |
| **18.** | **在户外时的个人安危**  **Your general safety when out of your home?** | **3** | **2** | **1** | **0** |
| **19.** | **在过去的一个月内， 您多经常因为视力而不能够做您想做的事？**  **In the past month, how often has your eyesight**  **stopped you doing the things you want to do?** | **3** | **2** | **1** | **0** |
| **20.** | **在过去的一个月内，您多经常因为视力问题而需要他人的帮助？**  **In the past month, how often have you needed**  **help from other people because of your eyesight?** | **3** | **2** | **1** | **0** |

若有佩戴眼镜、隐形眼镜或使用放大镜， 请根据您使用它们时的视力回答以下问题。回想过去的一个月，您的视力所带给您的感受。

Please answer about YOUR eyesight with GLASSES, CONTACT LENSES or MAGNIFIERS, if you use them.

Think about how YOUR eyesight has made you FEEL in the PAST MONTH.

|  | | 完全没有  Not at all | 有些时候  A little of time | 相当多的时候  A fair amount of time | 非常多的时候  A lot of time |
| --- | --- | --- | --- | --- | --- |
| **21.** | **您有没有因为您的视力而感到尴尬?**  **Have you felt embarrassed because of**  **your eyesight?** | **3** | **2** | **1** | **0** |
| **22.** | **您有没有因为您的视力而感到烦躁?**  **Have you felt frustrated or annoyed**  **because of your eyesight?** | **3** | **2** | **1** | **0** |
| **23.** | **您有没有因为您的视力而感到寂寞或孤立?**  **Have you felt lonely or isolated because of**  **your eyesight?** | **3** | **2** | **1** | **0** |
| **24.** | **您有没有因为您的视力而感到难过或情绪低落?**  **Have you felt sad or low because of your**  **Eyesight?** | **3** | **2** | **1** | **0** |
| **25.** | **在过去的一个月，您多经常担心您的视力会变差？**  **In the past month, how often have you**  **worried about your eyesight getting worse?** | **3** | **2** | **1** | **0** |
| **26.** | **在过去的一个月，您多经常因您的视力而担心您应付日常生活的能力？**  **In the past month how often has your**  **eyesight made you concerned or worried**  **about coping with everyday life?** | **3** | **2** | **1** | **0** |
| **27.** | **您有没有因为您的视力而感到自己是个麻烦或负担?**  **Have you felt like a nuisance or a burden**  **because of your eyesight?** | **3** | **2** | **1** | **0** |
| **28.** | **在过去的一个月，您的视力对您的生活造成了多大的干扰？**  **In the past month, how much has your**  **eyesight interfered with your life in general?** | **3** | **2** | **1** | **0** |

请检查您已回答所有的问题。谢谢！

Please check that you have answered all the questions and Thank you!
